# Highly diverse anaerobe-predominant vaginal microbiota among HIV-infected pregnant women in Zambia

**DOI:** 10.1371/journal.pone.0223128

**Published:** 2019-10-02

**Authors:** Joan T. Price, Bellington Vwalika, Marcia Hobbs, Julie A. E. Nelson, Elizabeth M. Stringer, Fei Zou, Katelyn J. Rittenhouse, Andrea Azcarate-Peril, Margaret P. Kasaro, Jeffrey S. A. Stringer

**Affiliations:** 1 Division of Global Women’s Health, Department of Obstetrics and Gynecology, University of North Carolina at Chapel Hill School of Medicine, Chapel Hill, North Carolina, United States of America; 2 Department of Obstetrics and Gynaecology, University of Zambia School of Medicine, Lusaka, Zambia; 3 UNC Global Projects Zambia, Lusaka, Zambia; 4 Division of Infectious Diseases, Department of Medicine, University of North Carolina at Chapel Hill School of Medicine, Chapel Hill, North Carolina, United States of America; 5 Department of Microbiology and Immunology, University of North Carolina at Chapel Hill School of Medicine, Chapel Hill, North Carolina, United States of America; 6 Department of Biostatistics, University of North Carolina Gillings School of Global Public Health, Chapel Hill, North Carolina, United States of America; 7 Microbiome Core Facility, University of North Carolina at Chapel Hill, Chapel Hill, North Carolina, United States of America; University of Cape Town, SOUTH AFRICA

## Abstract

Vaginal dysbiosis has been shown to increase the risk of some adverse birth outcomes. HIV infection may be associated with shifts in the vaginal microbiome. We characterized microbial communities in vaginal swabs collected between 16–20 gestational weeks in the Zambian Preterm Birth Prevention Study to investigate whether HIV and its treatment alter the microbiome in pregnancy. We quantified relative abundance and diversity of bacterial taxa by whole-genome shotgun sequencing and identified community state types (CST) by hierarchical clustering. Associations between exposures—HIV serostatus (HIV+ vs HIV-) and preconceptional ART (ART+ vs ART-)—and microbiome characteristics were tested with rank-sum, and by linear and logistic regression, accounting for sampling by inverse-probability weighting. Of 261 vaginal swabs, 256 (98%) had evaluable sequences; 98 (38%) were from HIV+ participants, 55 (56%) of whom had preconceptional ART exposure. Major CSTs were dominated by: *L*. *crispatus* (CST 1; 17%), *L*.*] iners* (CST 3; 32%), *Gardnerella vaginalis* (CST 4-I; 37%), *G*. *vaginalis* & *Atopobium vaginae* (CST 4-II; 5%), and other mixed anaerobes (CST 4-III; 9%). *G*. *vaginalis* was present in 95%; mean relative abundance was higher in HIV+ (0.46±0.29) compared to HIV- participants (0.35±0.33; rank-sum p = .01). Shannon diversity was higher in HIV+/ART+ (coeff 0.17; 95%CI (0.01,0.33), p = .04) and HIV+/ART- (coeff 0.37; 95%CI (0.19,0.55), p < .001) participants compared to HIV-. Anaerobe-dominant CSTs were more prevalent in HIV+/ART+ (63%, AOR 3.11; 95%CI: 1.48,6.55, p = .003) and HIV+/ART- (85%, AOR 7.59; 95%CI (2.80,20.6), p < .001) compared to HIV- (45%). Restricting the comparison to 111 women in either CST 3 (*L*. *iners* dominance) or CST 1 (*L*. *crispatus* dominance), CST 3 frequency was similar in HIV- (63%) and HIV+/ART- participants (67%, AOR 1.31; 95%CI: (0.25,6.90), p = .7), but higher in HIV+/ART+ (89%, AOR 6.44; 95%CI: (1.12,37.0), p = .04). Pregnant women in Zambia, particularly those with HIV, had diverse anaerobe-dominant vaginal microbiota.

## Introduction

The vaginal microbial environment plays an important role in sexual and reproductive health outcomes. In contrast to the gut, where low microbial diversity is associated with disease, most vaginal microbial communities defined in Western studies are dominated by one of four aerobic *Lactobacillus* species: *L*. *crispatus* (Community State Type [CST] 1), *L*. *gasseri* (CST 2), *L*. *iners* (CST 3), or *L*. *jensenii* (CST 5).[1] Another community state type (CST 4), characterized by predominance of anaerobic species like *Gardnerella vaginalis* and other bacteria implicated in clinical bacterial vaginosis, has been linked to increased susceptibility to sexually transmitted infections and HIV,[2, 3] perioperative and peripartum pelvic infections,[4, 5] and infertility.[6] Anaerobe species can disturb the vaginal epithelial barrier integrity, incite an inflammatory response, and impede wound healing.[7, 8] Conversely, relative abundance of *Lactobacillus* species in the vagina causes increased production of lactic acid and low pH, which is protective against a range of infections.[9]

During pregnancy, vaginal microbiota dominated by anaerobes and devoid of *Lactobacillus* have been associated with adverse birth outcomes such as preterm birth (PTB).[10, 11] Even within the *Lactobacillus* genus, predominance of the *L*. *iners* species seems to carry higher risk of vaginal dysbiosis,[12] acquisition of HIV,[13] and PTB[14] compared to other species (especially *L*. *crispatus*). Maternal HIV infection, which complicates as many as 1 in 4 pregnancies in Southern Africa,[15] can increase the risk of preterm delivery by as much as half.[16] Counterintuitively, HIV-infected women who initiate antiretroviral therapy (ART) before pregnancy may have even higher rates of preterm delivery than those who start ART during pregnancy.[17] The biological mechanism(s) linking the virus and its treatment to PTB remain poorly understood.

Few studies to date have characterized the vaginal microbiota of peripartum African women,[18–20] and limited data exist comparing the vaginal flora between HIV-infected and uninfected gravidas,[21] or by timing of ART initiation.[22] We hypothesize that maternal HIV infection and ART exposure may be associated with measurable differences in vaginal microbial communities during pregnancy. In this baseline cross-sectional analysis, we characterize the vaginal microbiota of participants in an ongoing pregnancy cohort in Zambia, and investigate whether HIV and its treatment may be associated with alterations of the microbiome during pregnancy.

## Methods

### Cohort

The Zambian Preterm Birth Prevention Study (ZAPPS; ClinicalTrials.gov Identifier: NCT02738892) is a prospective antenatal cohort underway at the Women and Newborn Hospital of the University Teaching Hospitals (UTH) in Lusaka. Participants are enrolled prior to 24 weeks’ gestation and receive comprehensive antenatal care, laboratory testing, and routine ultrasounds at first encounter to establish gestational age and again in the third trimester to monitor fetal growth. Participants also undergo scheduled biological specimen collection for long-term storage and protocol-related testing.

We defined our exposure as a 3-level variable that characterized participants by HIV serostatus and—among those infected—by timing of ART exposure. We determined HIV infection at cohort enrollment by screening all participants using the Determine HIV-1/2 Ag/Ab Combo test (Alere Inc., Waltham, MA) and confirming positive cases with SD Bioline 3.0 test (SD Biostandard Diagnostics, India). Timing of ART exposure—defined as either preconceptional or not preconceptional—was determined by review of medical records and viral load assays (i.e., undetectable viral load consistent with preconceptional ART exposure). Viral load assays were conducted using the Abbott RealTime HIV-1 Assay (Abbott Molecular, Des Plaines, IL). In participants with unknown ART start date and detectable viral load, we tested contemporaneous plasma samples for ART drug concentration via liquid chromatography/mass spectrometry.[23–25]

The University of Zambia Biomedical Research Ethics Committee (reference number: 016-04-14) and the University of North Carolina Institutional Review Board (study number: 14–2113) each granted approval to conduct the ZAPPS study and for protocol-related specimen testing. Participants in ZAPPS provided individual written informed consent in the language of their choice (English, Nyanja, or Bemba) before undergoing any study procedures.

### Specimen collection and sample selection

In the ongoing ZAPPS cohort, trained research nurses collect mid-vaginal secretions using dry polyester swabs on all study participants at enrollment prior to 24 weeks’ gestation. Specimens are processed and then stored on-site in temperature-controlled freezers at -80°C. ZAPPS biological specimens are shipped periodically from the field site in Zambia to our lab in Chapel Hill, NC for testing and archiving. This analysis includes specimens obtained from the first such shipment (May 2017), which included vaginal swabs obtained between 16–20 gestational weeks from 461 participants. To maximize available budget, we used a double sampling technique that selected specimens from all HIV-infected participants and all of those who delivered prior to 37 weeks gestation. Among the remainder (HIV-uninfected women with either term birth or with unknown birth outcome), we selected specimens at random at a proportion dictated by available resources.

### DNA isolation

We performed whole genome shotgun (WGS) sequencing of bacterial DNA extracted from vaginal swabs to quantify relative abundance and diversity of bacterial taxa. Vaginal swab samples were transferred to a 2 mL tube containing 200 mg of ≤ 106 μm glass beads (Sigma, St. Louis, MO) and 0.3 mL of Qiagen ATL buffer (Valencia, CA), supplemented with 20 mg/mL lysozyme (Thermo Fisher Scientific, Grand Island, NY). The suspension was incubated at 37°C for 1 hour with occasional agitation. Subsequently, the suspension was supplemented with 600 IU of Qiagen proteinase K and incubated at 60°C for 1 h. Finally, 0.3 mL of Qiagen AL buffer was added and a final incubation at 70°C for 10 minutes was carried out. Bead beating was then employed for 3 minutes in a Qiagen TissueLyser II at 30 Hz. After a brief centrifugation, supernatants were aspirated and transferred to a new tube containing 0.3 mL of ethanol. DNA was purified using a standard on-column purification method with Qiagen buffers AW1 and AW2 as washing agents, and eluted in 10mM Tris (pH 8.0).

### Whole Genome Shotgun (WGS) sequencing

1 ng of genomic DNA was processed using the Nextera XT DNA Sample Preparation Kit (Illumina). Target DNA was simultaneously fragmented and tagged using the Nextera Enzyme Mix containing transposome that fragments the input DNA and adds the bridge PCR (bPCR)-compatible adaptors required for binding and clustering in the flowcell. Next, fragmented and tagged DNA was amplified using a limited-cycle PCR program. In this step index 1(i7) and index 2(i5) were added between the downstream bPCR adaptor and the core sequencing library adaptor, as well primer sequences required for cluster formation. The thermal profile for the amplification had an initial extension step at 72°C for 3 min and initial denaturing step at 95°C for 30 sec, followed by 12 cycles of denaturing of 95°C for 10 seconds, annealing at 55°C for 30 seconds, a 30 seconds extension at 72°C, and final extension for 5 minutes at 72°C. The DNA library was then purified using Agencourt® AMPure® XP Reagent. Each sample was quantified and normalized prior to pooling. The DNA library pool was loaded on the Illumina platform reagent cartridge and on the Illumina instrument.

### Bioinformatic data analysis

Sequencing output from the Illumina HiSeq platform was converted to FASTQ format and demultiplexed using Illumina Bcl2Fastq 2.18.0.12 conversion software (Illumina). Quality control of the demultiplexed sequencing reads were verified with FastQC software (Babraham Institute, Cambridge, UK). The resulting paired-end reads were aligned with Bowtie2[26] against the host reference (Human Hg19 if human) and all aligning human reads were eliminated. Paired-end reads were joined with VSEARCH 1.10.2.[27] The resulting single-end reads were again aligned against the human reference with Bowtie2 retaining all reads that did not align. Estimates of taxonomic composition, gene family, path abundance, and path coverage were produced from the remaining reads using the HUMAnN2 pipeline.

Bacterial taxa with fewer than 5 reads were treated as absent in that sample. To identify major CSTs in our population, agglomerative hierarchical clustering analysis with Bray-Curtis dissimilarity distance was applied to the proportional compositions of the 29 predominant taxa, each with a sum of relative abundance across study subjects of at least 10%. Shannon diversity index—a measure of species richness and evenness—was calculated for each sample as a sum of each individual species’ proportional abundance multiplied by the natural logarithm of this same proportion.[28]

### Statistical data analysis

We analyzed baseline demographic data of the sub-study cohort, calculating median and interquartile range (IQR) for each continuous variable, and frequency and percentage for each categorical variable. We analyzed differences in continuous independent variables between our exposure groups by Kruskal-Wallis two-sample statistic.

We investigated associations between our exposure groups and outcomes: mean relative abundances of major taxa, mean Shannon diversity indices, and CSTs. Differences in mean bacterial abundances and in diversity between HIV-uninfected versus infected participants with and without preconceptional ART exposure were analyzed via linear regression, accounting for multiple comparisons with Bonferroni correction. We evaluated the association between our exposure variable and CSTs using logistic regression. Because birth outcome data from the ZAPPS cohort were not yet finalized at the time of this analysis, we did not analyze them here. We applied inverse probability sampling weights to univariate and multivariable analyses to account for unequal sampling technique, with robust standard errors computed by the linear variance estimator.[29] All regression models were adjusted for potential confounding due to baseline characteristics that varied by exposure group.

The sample size for this study was determined by available biological specimens and budget. We did not perform a formal sample size calculation prior to undertaking this descriptive research. Analyses were performed using R version 3.5.0 microbiome[30] and phyloseq[31] packages, and Stata version 14 (College Station, TX, USA).

## Results

### Baseline characteristics

Between August 2015 and September 2017, 1450 participants were enrolled in ZAPPS.[32] At the time of this analysis, vaginal swabs collected between 16–20 gestational weeks were available for 461 participants. We performed WGS on 261 (57%) of the available swabs, which represented vaginal swabs from all HIV-infected participants (n = 99/99), all HIV-uninfected participants who went on to deliver preterm (n = 28/28), and a random sample of HIV-uninfected participants who delivered at term (n = 119/259; 46%) or who had an unknown delivery outcome at the time of analysis (15/75; 20%). Of the tested swabs, 256 (98%) had evaluable bacterial sequence data (i.e., at least 5 reads of at least one bacterial taxon), 98 (38%) were from HIV-infected participants, 55 (56%) of whom had initiated ART before conception, and 41 (42%) of whom had not. Two HIV-infected participants had undetermined timing of ART initiation (Table 1).

**Table 1:** Baseline characteristics of participants with evaluable bacterial sequences, N = 256.

Characteristic	Overall		HIV-		HIV+ *preconceptional ART*		HIV+ *no preconceptional ART*	
	N or median	% or IQR	N or median	% or IQR	N or median	% or IQR	N or median	% or IQR
Overall^a^	256	100	158	61.7	55	21.5	41	16.0
Age, years	27	(22,32)	25	(22,31)	31	(27,33)	25	(22,29)
<20	24	9.7	20	13.1	0	0	4	10.0
20–34	194	78.5	114	74.5	45	83.3	35	87.5
≥35	29	11.7	19	12.4	9	16.7	1	2.5
missing	7		5		1		1	
Marital status								
Married or cohabiting	208	82.9	128	81.5	46	85.2	34	85.0
missing	3		1		1		1	
Smoking	1	0.4	0	0	0	0	1	2.5
missing	4		1		2		1	
Alcohol use	25	10.0	13	8.3	6	11.3	6	15.0
missing	4		1		2		1	
BMI, kg/m^2^	23.9	(21.8,27.0)	24.2	(21.9,27.9)	24.5	(22.1,26.6)	22.3	(20.3,25.4)
<18.5	9	3.6	5	3.3	1	1.9	3	7.5
18.5–30	201	81.4	121	79.1	45	83.3	35	87.5
>30	37	15.0	27	17.7	8	14.8	2	5.0
missing	7		5		1		1	
Gravidity	3	(1,4)	2	(1,3)	3	(2,4)	3	(2,3)
Parity	1	(0,2)	1	(0,2)	2	(1,3)	1	(1,2)
Prior PTB								
0 (nulliparous)	71	27.7	55	34.8	6	10.9	9	22.0
0 (parous)	107	41.8	61	38.6	31	56.4	15	36.6
1 or more	78	30.5	42	26.6	18	32.7	17	41.5
EGA at collection, weeks	18	(17,19)	18	(17,19)	18	(17,19)	18	(17,19)
Multiple gestation	8	3.2	5	3.2	1	1.8	2	4.9
Short Cervix < 2.5 cm	6	2.8	2	1.5	2	4.1	2	6.1
missing	37		23		6		8	
Hemoglobin < 10.5 g/dL	38	21.6	20	19.2	7	17.5	11	34.4
missing	78		54		15		9	
Syphilis reactive	18	7.3	9	5.9	5	9.3	4	10.0
missing	7		5		1		1	
Abnormal urinalysis	11	4.3	6	3.8	2	3.6	3	7.3
missing	6		2		3		0	
Vaginal washing	81	34.8	54	35.8	13	31.7	14	34.2
missing	21		7		14		0	
Sexual intercourse <24 hours	69	29.4	53	34.6	9	22.0	7	17.1
missing	19		5		14		0	
Vaginal pH, mean (±SD)	5.52	0.61	5.46	0.64	5.54	0.58	5.73	0.46
missing	10		5		3		2	
HIV diagnosis timing								
During pregnancy					0	0	34	82.9
<5 years before pregnancy					44	80.0	3	7.3
5+ years before pregnancy					11	20.0	4	9.8
HIV RNA viral copies/μL					0	(0,0)	12361	(2956,31358)
<40 (undetectable)					49	89.1	0	0
CD4 cells/mL, n = 19					500	(426,654)	335	(242,475)
CD4 <200 cells/mL					1	7.7	1	16.7
missing					42		35	

IQR, interquartile range; BMI, body mass index; PTB, preterm birth; EGA, estimated gestational age

^a^ Except for the overall columns, this table excludes 2 HIV-infected participants with unknown ART timing

The median age of participants at enrollment was 27 (IQR 22,32); HIV-infected participants who initiated ART prior to conception were older (median: 31 years) than uninfected (median: 25 years) and HIV-infected without preconceptional ART (median: 25 years; *p* < .001). HIV-infected participants without preconceptional ART exposure had lower BMI (median: 22.3 kg/m^2^) than both uninfected participants (median: 24.2 kg/m^2^) and infected participants who had initiated ART prior to conception (median 24.5 kg/m^2^; p = .04). Parity differed by HIV serostatus and ART exposure: HIV-infected participants with preconceptional ART exposure had higher parity (median: 2) compared to those without preconceptional ART exposure (median: 1) and HIV-uninfected participants (median: 1; p = .03). Participants with preconceptional ART exposure had lower viral loads at enrollment (median 0 versus 12,361 copies/μL, p < .001) and trended toward higher CD4 counts (median 500 versus 335; p = 0.22) than those without preconceptional ART exposure.

### Taxonomic prevalence, community states, and diversity

**Fig 1:**
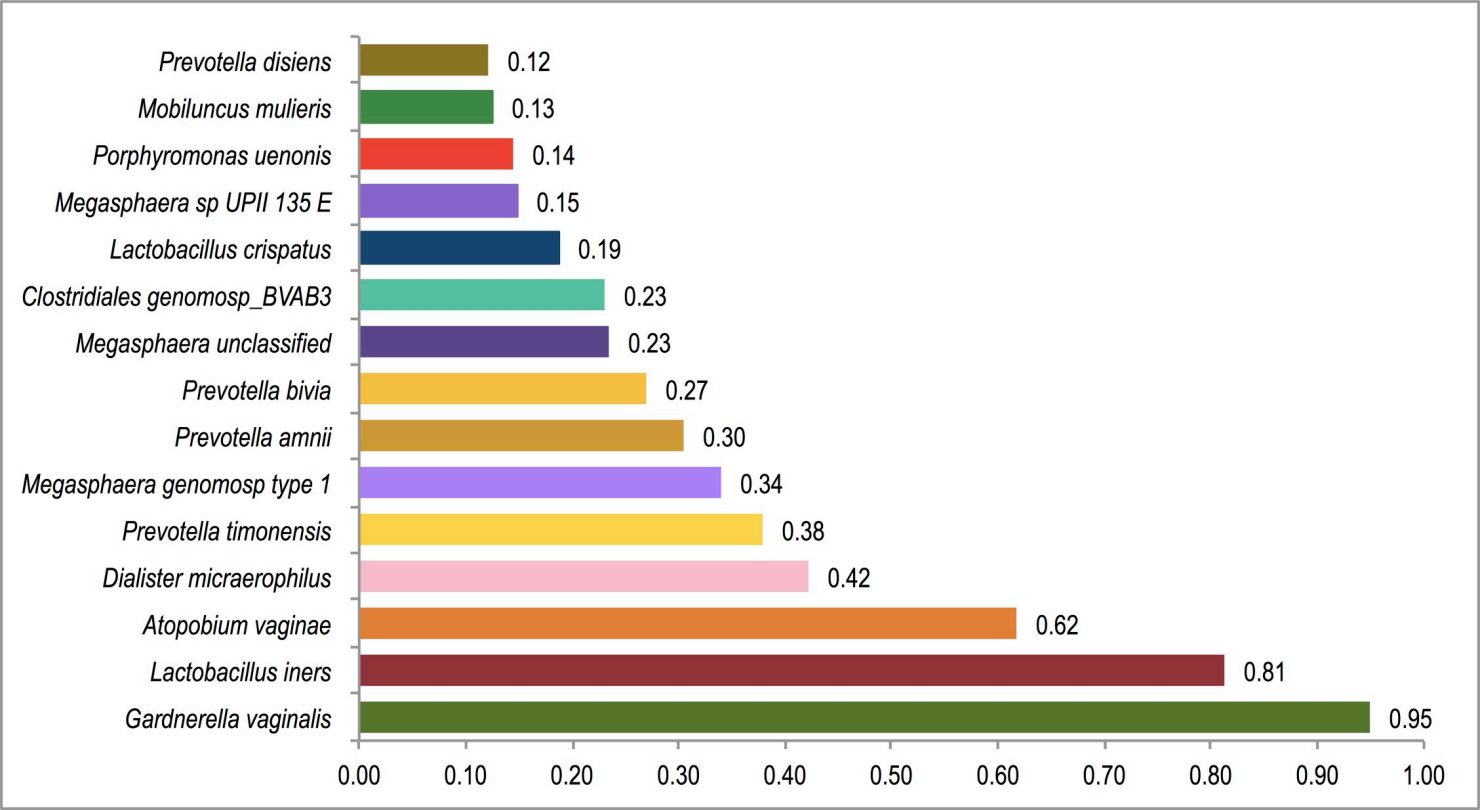
Prevalence of bacterial taxa in pregnant ZAPPS cohort, n = 256. Overall prevalence of each of the 15 most common bacterial taxa across all participants, with *Gardnerella vaginalis*, *Lactobacillus iners*, and *Atopobium vaginae*, each isolated in over half of all specimens.

Ninety-eight bacterial taxa were detected in the vaginal specimens included in this analysis. The most prevalent bacterial species was *Gardnerella vaginalis*, which was identified in 95% (n = 243) of samples (Fig 1). The mean relative abundance of *G*. *vaginalis* was higher among HIV-infected (0.46±0.29) compared to uninfected participants (0.35±0.33; rank-sum p = .01).

**Fig 2:**
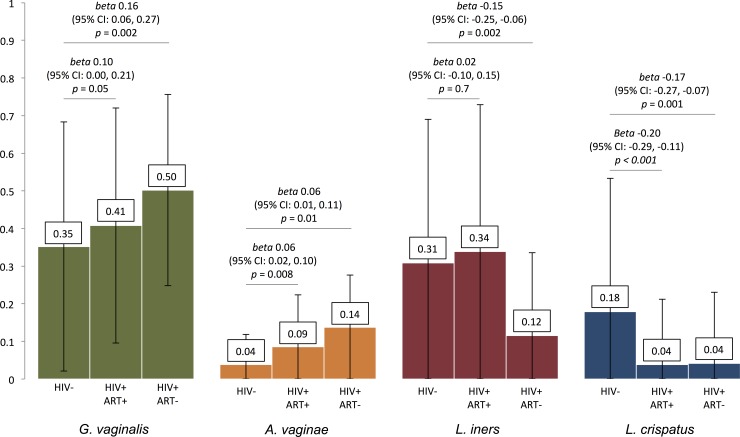
Differences in relative abundance of 4 key bacterial taxa by HIV serostatus and ART timing. Relative proportions with regression coefficients and 95% confidence intervals of *Gardnerella vaginalis*, *Atopobium vaginae*, *Lactobacillus iners*, and *Lactobacillus crispatus* among HIV-infected women with (ART+) and without (ART-) preconceptional exposure to antiretroviral therapy compared to HIV-uninfected women. HIV-infected women with and without preconceptional ART exposure demonstrate higher abundance of *Atopobium vaginae* and lower *Lactobacillus crispatus*, whereas those without preconceptional ART exposure have higher *Gardnerella vaginalis* abundance and lower *Lactobacillus crispatus*. All regression coefficients adjusted for maternal age, body mass index, and parity. ART, antiretroviral therapy.

In regression analyses of relative abundances of bacterial taxa by HIV and ART exposure status (adjusted for maternal age, BMI, and parity), HIV-infected women without preconceptional ART exposure had higher relative abundance of *G*. *vaginalis* compared to uninfected women (coeff 0.16, 95% CI 0.06,0.27, corrected p = .01) (Fig 2). Both HIV-infected participants with preconceptional ART exposure (coeff 0.06, 95% CI 0.02,0.10, corrected p = .02) and those without it (coeff 0.06, 95% CI 0.01,0.11, corrected p = .04) had higher relative abundance of *Atopobium vaginae* compared to HIV-uninfected participants. In contrast, relative abundances of key *Lactobacillus* species were lower among HIV-infected compared to uninfected participants. Relative abundance of *L*. *crispatus* was lower among both HIV-infected participants with preconceptional ART exposure (coeff -0.20, 95% CI -0.29,-0.11, corrected p < .001) and those without (coeff -0.17, 95% CI -0.27,-0.07, corrected p = .008). HIV-infected women without preconceptional ART exposure had lower relative abundance of *L*. *iners* (coeff -0.15, 95% CI -0.25,-0.06, corrected p = .008) compared to HIV-uninfected women. Relative abundances of other bacterial taxa were similar between exposure groups.

**Fig 3:**
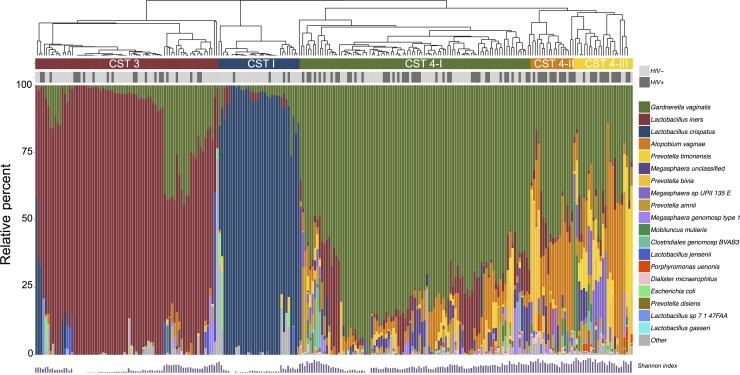
Bacterial diversity plot of major bacterial taxa and identified community states in ZAPPS. Diversity plot demonstrating the relative abundance of the most common bacterial species identified across all specimens (n = 256) and the results of hierarchical clustering into 5 major community state types dominated by: *Lactobacillus crispatus* (CST I), *Lactobacillus iners* (CST 3), *Gardnerella vaginalis* (CST 4-I), *Gardnerella vaginalis* & *Atopobium vaginae* (CST 4-II), and mixed anaerobes (CST 4-III). CST, community state type.

We identified 5 major clustered community states in our samples dominated by: *L*. *crispatus* (CST 1; n = 34/256, 17% weighted), *L*. *iners* (CST 3; n = 77/256, 32% weighted), *G*. *vaginalis* (CST 4-I; n = 97/256, 37% weighted), *G*. *vaginalis* & *A*. *vaginae* (CST 4-II, n = 18/256, 5% weighted), and mixed anaerobes (CST 4-III, n = 25/256, 9% weighted) (Fig 3). There was substantial prevalence of *G*. *vaginalis* in samples within the *L*. *iners*-dominant CST 3: the mean relative abundance of *L*. *iners* across specimens clustered in CST 3 was 0.81 (SD±0.19); the second most common species was *G*. *vaginalis* (0.11±0.14).

**Fig 4:**
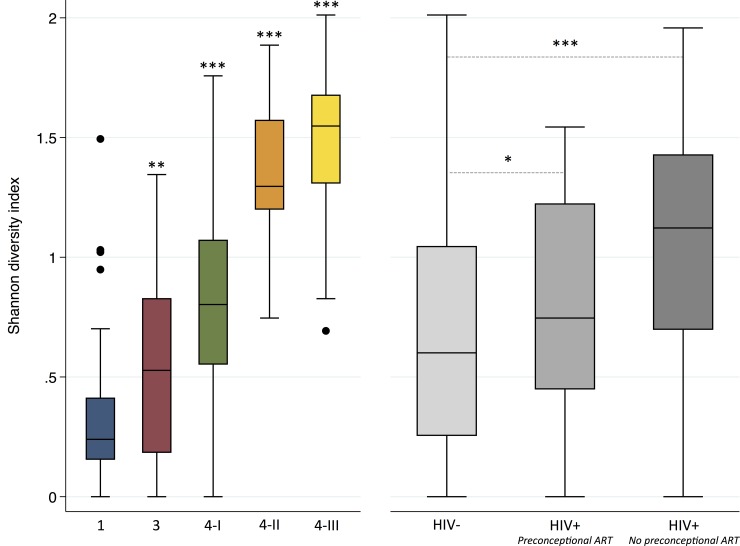
**Shannon diversity indices by community state type (panel A) and by HIV serostatus and timing of initiation of antiretroviral therapy (panel B) in pregnant ZAPPS cohort.** Species diversity of the vaginal microbiome increases across CSTs; *Lactobacillus*-dominant CSTs 1 and 3 have the lowest diversity and anaerobe-dominant CSTs have highest diversity. Diversity is higher among HIV-infected participants with and without preconceptional ART exposure compared to uninfected participants. *p* values calculated by linear regression of Shannon diversity index between individual CSTs compared to CST1 (panel A) and between exposure groups (panel B) All analyses weighted for sampling. * p *=* .04, ** p = .001, *** p < .001 CST, community state type; ART, antiretroviral therapy.

Shannon diversity indices varied significantly by CST. Microbiota in CST 1, dominated by *L*. *crispatus*, had the lowest diversity, and those in CST 3, 4-I, 4-II, and 4-III each demonstrated incrementally higher diversity (Fig 4A; Table 2). Microbial diversity also varied by HIV serostatus and ART exposure: diversity was lower in HIV-uninfected participants (0.66±0.47) compared to infected participants with preconceptional ART exposure (0.78±0.47, p = .04) and those without (1.07±0.49, p < .001) (Fig 4B; Table 2).

**Table 2:** Mean Shannon diversity indices (MSDI) in 5 major community state types and by HIV serostatus and timing of antiretroviral therapy exposure.

	N	Raw %	MSDI (±SD)	Unadjusted^a^			Adjusted^a^		
				coeff	95% CI	*p*	coeff	95% CI	*p*
*Community state type* ^b^									
**CST 1**	34	13.6%	0.35 (±0.34)	*ref*			*ref*		
**CST 3**	77	30.7%	0.46 (±0.36)	0.22	0.07,0.37	0.005	0.25	0.10,0.40	0.001
**CST 4-I**	97	38.7%	0.82 (±0.35)	0.49	0.36,0.62	< .001	0.51	0.38,0.64	< .001
**CST 4-II**	18	7.2%	1.33 (±0.26)	1.02	0.85,1.18	< .001	1.03	0.87,1.18	< .001
**CST 4-III**	25	10.0%	1.45 (±0.34)	1.16	0.99,1.33	< .001	1.17	0.99,1.34	< .001
*HIV serostatus and ART exposure*^*c*^									
**HIV-**	158	62.2%	0.66 (±0.47)	*ref*			*ref*		
**HIV+** *Preconceptional ART*	55	21.7%	0.78 (±0.47)	0.09	-0.06, 0.24	0.2	0.17	0.01,0.33	0.04
**HIV+** *No preconceptional ART*	41	16.1%	1.07 (±0.49)	0.38	0.21, 0.55	< .001	0.37	0.19,0.55	< .001

CST, community state type; MSDI, mean Shannon diversity index; CI, confidence interval; coeff, coefficient of linear regression; ref, referent category; ART, antiretroviral therapy

^a^ Coefficients and *p*-values calculated by linear regression of Shannon Index of each CST compared to CST 1 or ART timing compared to HIV-negative, weighted for sampling. Multivariable analyses adjusted for maternal age, BMI, and parity

^b^ Excludes 5 in minor CSTs

^*c*^ Excludes 2 HIV-infected participants with undetermined ART timing

**Fig 5:**
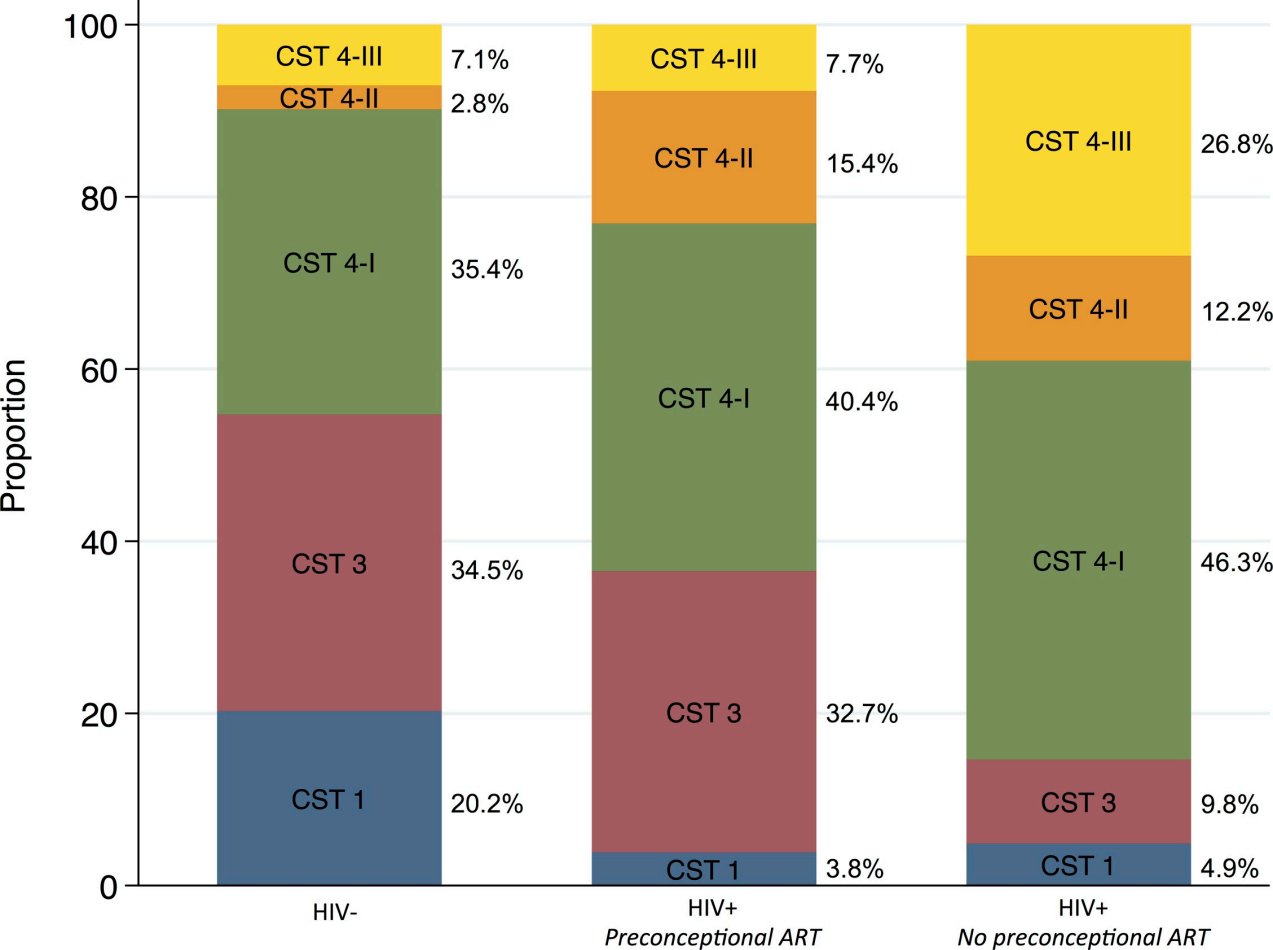
Prevalence of community state types by HIV serostatus and timing of initiation of antiretroviral therapy in ZAPPS. Anaerobe-dominant community state types were most prevalent among HIV-infected participants, both with and without preconceptional ART exposure. Comparing *Lactobacillus*-dominated CSTs only, HIV-infected participants with preconceptional ART exposure had higher relative abundance of CST 3 over CST 1 compared to uninfected participants. CST, community state type; ART, antiretroviral therapy.

Strict anaerobe-dominant CSTs (i.e., CST 4-I, 4-II, & 4-III) were more prevalent among HIV-infected participants, including both those with preconceptional ART exposure (63% weighted, AOR 3.11; p = .003) and without (85% weighted, AOR 7.59; p < .001), compared to HIV-uninfected participants (45% weighted) (Fig 5; Table 3). Restricting the comparison to 111 women in either CST3 (*L*. *iners* dominance) or CST 1 (*L*. *crispatus* dominance), the frequency of CST3 was similar in HIV-uninfected (63% weighted) and HIV-infected participants without preconceptional ART exposure (67% weighted), but significantly higher with preconceptional ART exposure (89% weighted, AOR 6.44; p = .04).

**Table 3:** Prevalence of community states types by HIV status and timing of antiretroviral therapy exposure, n = 249.

	N^a^	CST I (*L*. *crispatus*)		CST 3 (*L*. *iners*)		CST 4-I (*G*. *vag*)		CST 4-II (*G*. *vag + A*. *vag*)		CST 4-III (Mixed spp.)		Anaerobe- vs. *Lactobacillus*-dominant *CST 4 vs CST 1–3*			*L*. *iners* vs. *L*. *crispatus* *CST 3 vs*. *1*		
		N	% ^b^	N	% ^b^	N	% ^b^	N	% ^b^	N	% ^b^	OR	95% CI	*p* ^c^	OR	95% CI	*p* ^c^
**HIV-**	156	30	20.2	56	34.5	55	35.4	5	2.8	10	7.1	*ref*			*ref*		
**HIV+** *Preconceptional ART*	52	2	3.8	17	32.7	21	40.4	8	15.4	4	7.7	3.11	1.48,6.55	0.003	6.44	1.12,37.0	0.04
**HIV+** *No preconceptional ART*	41	2	5.0	4	9.8	19	46.3	5	12.2	11	26.9	7.59	2.80,20.6	< .001	1.31	0.25,6.90	0.7

CST, community state type; OR, odds ratio; ART, antiretroviral therapy

^a^ Analyses exclude 5 in minor CSTs, and 2 HIV-infected participants with unknown ART timing

^b^ Relative percent weighted for sampling

^c^ Odds ratios and *p*-values calculated by logistic regression of CST compared to HIV-negative, adjusting for maternal age, BMI, and parity, and weighted for sampling

## Discussion

We have characterized the vaginal microbiota of a cohort of pregnant women with high HIV prevalence in urban Zambia, building on a sparse body of literature that describes vaginal microbial communities among African women in the peripartum period.[18, 19, 21] In our study, whole genome sequencing of early second trimester vaginal specimens clustered into 5 major community state types, many of which demonstrated high diversity and prevalence of the anaerobic bacteria *Gardnerella vaginalis* and *Atopobium vaginae*. Maternal HIV infection was associated with greater microbial diversity and with anaerobe-dominant vaginal community clusters. Compared to HIV-uninfected participants, infected women had lower relative abundance of *Lactobacillus crispatus*, and participants with preconceptional ART exposure had higher prevalence of vaginal microbial communities dominated by *Lactobacillus iners*.

We present evidence of variation in the composition of vaginal microbiota by HIV serostatus in pregnancy. Numerous studies in non-pregnant women in sub-Saharan Africa have shown diverse anaerobe-dominant vaginal microbiota are associated with an increased risk of HIV acquisition, reduced efficacy of 1% tenofovir gel prophylaxis, and more viral shedding among those infected, while *Lactobacillus crispatus* may exert a protective effect.[2, 33–36] In a case-control study of mother-to-child HIV transmission in Burkina Faso, pregnant women who transmitted the virus had higher abundance of *Gardnerella vaginalis* and *Prevotella* species and lower abundance of the phylum that includes *Lactobacillus* species.[20] Finally, a recent longitudinal study among postpartum Malawian women found that *Lactobacillus*-deficient microbiota was the most common vaginal microbial composition up to a year postpartum, and reported trends toward higher prevalence of *Mycoplasma hominis* and *Clostridiales* BVAB3 among HIV-infected compared to uninfected women.[18] We cannot conclude from this research that HIV infection is causally associated with alterations in the vaginal microbiome, and we acknowledge the possibility that our results instead reflect evidence of the same vaginal microbial composition associated with HIV acquisition. Future cohort studies should investigate longitudinal stability of the vaginal microbiome to further characterize the effect of HIV through pregnancy and the postpartum period.

In ZAPPS, HIV-infected participants who initiated ART before pregnancy had higher odds of microbiota dominated by *L*. *iners* over *L*. *crispatus* when compared to uninfected women and relative abundance of *L*. *iners* was lowest among those without preconceptional ART. A cohort study in the UK found women with preconceptional exposure to ART had higher abundance of certain anaerobic species including *Atopobium vaginae.[22]* However, the measured effect of preconceptional ART exposure on the vaginal microbiome could be attributed to confounding by indication: women with longer duration of HIV infection or more advanced disease are more likely to have initiated ART before pregnancy. Our results were limited by a relatively small number of participants without preconceptional ART exposure such that we did not directly compare ART exposure groups nor could we compare the sub-groups of women who newly started ART during pregnancy to those who had no antenatal ART exposure at all. Again, a larger cohort with longitudinal sample collection could better investigate the effect of newly starting ART, and the relative effects of specific drug regimens, on the vaginal microbiome in pregnancy.

Bacterial vaginosis, a clinical diagnosis classically characterized by predominance of strictly anaerobic or aerotolerant bacterial species and a relative paucity of lactic acid-producing *Lactobacillus* species, has long been associated with adverse pregnancy outcomes.[11, 37] The recent availability of culture-independent molecular sequencing techniques has transformed the clinical diagnosis of bacterial vaginosis to a more granular classification of vaginal microbial communities. Ravel and colleagues published one of the first descriptions of vaginal microbiota among reproductive-aged women in the United States, using 16S ribosomal RNA amplicon sequencing and hierarchical clustering to characterize 4 CSTs (CST 1–3 and 5) each dominated by a different *Lactobacillus* species plus a fifth (CST 4) characterized by predominance of mixed anaerobic species and a relative paucity of *Lactobacillus*.[1] This latter microbially diverse community state was prevalent among just 10% of white women compared to nearly 40% of black and Hispanic women in Ravel’s American cohort. In our pregnant cohort in Zambia, with distributions weighted for sampling technique, over half of women during the second trimester were classified into one of three sub-groups of CST 4, whereas no comparable CST 5 (*L*. *jensenii*) was identified and only one participant was classified into a minor CST characterized by high relative abundance of *L*. *gasseri* (CST 2). Other minor CSTs identified in our study included one dominated by mixed *Porphyromonas* species (n = 1) and another by a mix of *Megasphaera*, *Prevotella*, and *Gardnerella* species (n = 3).

While *Lactobacillus* species were found in nearly all samples in our cohort, most were *L*. *iners* mixed with *Gardnerella vaginalis*; other *Lactobacillus* species were rare. Among postpartum women in Malawi, *Lactobacillus* species were also predominantly *L*. *iners* while *L*. *crispatus* was nearly entirely absent.[18] Kindinger and colleagues found that black women, who made up just 19% of their cohort in the UK, had higher prevalence of *L*. *iners* and diverse anaerobe-dominant CSTs compared to white women.[14] Yet while analysis of outcomes stratified by race and ethnicity showed no statistical difference between groups, 53% of white women who delivered at >34 gestational weeks had *L*. *crispatus* dominance (CST 1) compared to just 24% of black women. In a case-control study in the US in which 79 out of 90 (88%) of the study subjects were black, although the relative abundance of *Lactobacillus* species increased through pregnancy, no differences in microbiome composition were noted between women who ultimately had a spontaneous preterm delivery and those who delivered at term.[38] Inconsistent associations between vaginal microbiota and PTB risk by race in various studies may relate to population effects such as differential prevalence of nonmicrobiota-associated causes of PTB and a methodological reliance on comparisons between discrete community groupings (e.g., CSTs) instead of the relative frequencies of key taxa.[39] These findings suggest that the vaginal microbiota described in some studies as either “abnormal” or “healthy” may not universally predict the same risks or protective effects across races and regions, and that distilled definitions of community states may not adequately describe nuanced taxon-level microbial interactions or effects of other environmental and behavioral factors in individual women. The overwhelming prevalence of *Gardnerella vaginalis* in our cohort and in the Malawian cohort may indicate normal variation in vaginal microbial milieu rather than pathological imbalance.

We have not presented birth outcomes in this baseline cross-sectional analysis because final birth outcome data from the ZAPPS cohort have yet to be finalized. Once these final data are available, we anticipate a second manuscript investigating the relationships between vaginal microbiota and adverse birth outcomes. Nevertheless, the trends we have found in vaginal microbial composition between pregnant women with HIV and those without HIV may support published evidence of a higher risk of preterm delivery with maternal HIV infection, and with initiation of ART before pregancy.[16, 17] If PTB related to HIV and its treatment follows an inflammatory phenotype and is mechanistically associated with alterations in the composition of the vaginal microbiome, future studies could test the efficacy of treatment with anti-inflammatory progesterone supplementation, probiotics, or vaginal microbial transplant in this high-risk group.[40, 41]

Most studies characterizing the human microbiome employ 16S rRNA gene sequencing techniques,[42] which typically do not allow classification of taxa at the species level. By contrast, WGS uses random primers to sequence overlapping regions of a genome and can produce species-level data of bacteria present in a sample. Samples processed by WGS for microbiome analysis show enhanced detection of bacterial species, increased diversity detection, and improved accuracy of species detection.[43] As a limitation of WGS, our output describes relative abundances and not absolute bacterial loads. Future studies should investigate whether bacterial load data offer superior comparators between pregnant women with HIV and those without, and enable more accurate prediction of adverse birth outcomes than analyses comparing relative abundances only.

The key strength of our study is that we were able to precisely describe the vaginal microbiome in a pregnant population with high HIV prevalence, affording statistical power to detect differences in relative species abundance and community states by HIV serostatus and ART timing. In our overall cohort, the HIV prevalence is 24%,[32] which we enhanced by oversampling available vaginal swabs from HIV-infected participants and from a random sample of uninfected participants within the gestational window of interest. Although we adjusted our analyses to account for sampling technique, characterizing the microbiome of all participants might have represented a more accurate population-level description. Even so, our cohort represents a high-risk obstetric population seeking care in a prematurity study at the country’s university referral hospital such that its generalizability to a lower-risk obstetric population may be limited. Known regional and racial variations in both the composition of vaginal microbiota and their association with adverse birth outcomes further limit the global generalizability of findings from our cohort, a limitation that ultimately speaks to the need for expanded studies of the vaginal microbiome in Africa. Finally, despite evaluating numerous maternal factors for possible confounding on the relationship between our exposures and outcomes, other covariates not measured in our cohort, such as sexually transmitted infections beyond syphilis and HIV, undiagnosed inflammatory processes, and HLA allele type, may have contributed to residual confounding.

## Conclusion

In summary, we show that maternal HIV infection is associated with high microbial diversity and low relative abundance of *L*. *crispatus* species. Our study characterizes the vaginal microbiome in a pregnant African cohort, demonstrating an association between the vaginal microbiome, maternal HIV infection, and timing of ART exposure relative to conception. Future studies are needed to investigate if these observed differences could explain a mechanism underlying HIV-related PTB and an incremental risk with preconceptional ART.
